# Utilization of Multi-Immunization and Multiple Selection Strategies for Isolation of Hapten-Specific Antibodies from Recombinant Antibody Phage Display Libraries

**DOI:** 10.3390/ijms18061169

**Published:** 2017-05-31

**Authors:** Antti Tullila, Tarja K. Nevanen

**Affiliations:** VTT Technical Research Centre of Finland, Tietotie 2, FI-02150 Espoo, Finland; tarja.nevanen@vtt.fi

**Keywords:** phage display, fragment antigen-binding (Fab), recombinant antibody, high-throughput, hapten, mycotoxin

## Abstract

Phage display technology provides a powerful tool for the development of novel recombinant antibodies. In this work, we optimized and streamlined the recombinant antibody discovery process for haptens as an example. A multi-immunization approach was used in order to avoid the need for construction of multiple antibody libraries. Selection methods were developed to utilize the full potential of the recombinant antibody library by applying four different elution conditions simultaneously. High-throughput immunoassays were used to analyse the binding properties of the individual antibody clones. Different carrier proteins were used in the immunization, selection, and screening phases to avoid enrichment of the antibodies for the carrier protein epitopes. Novel recombinant antibodies against mycophenolic acid and ochratoxin A, with affinities up to 39 nM and 34 nM, respectively, were isolated from a multi-immunized fragment antigen-binding (Fab) library.

## 1. Introduction

Monoclonal and polyclonal antibodies obtained by immunization of animals are widely used as binding proteins in immunodiagnostic applications. However, their binding properties are dependent on the success of the immunization. Furthermore, the functionality of obtained polyclonal antibodies can vary between immunization batches, and hybridoma cell lines producing monoclonal antibodies may suffer from instability [1]. Recombinant antibody technology can circumvent these limitations. They can be developed against toxic and non-immunogenic target molecules and their heterologous production in bacterial cells is reproducible. Currently, the commonly used recombinant in vitro antibody display technologies include yeast [2], ribosome [3], mammalian cell [4], and phage display [5]. Of these technologies, phage display has become the most used in vitro method for recombinant antibody discovery.

Small molecules such as steroids, toxins, vitamins, drugs, and pollutants are important analytes in diagnostics. Typically, they have a size of <1000 g/mol, and due to their small size these haptens are not able to elicit immune response by themselves in vivo. However, when haptens are conjugated to carrier proteins the immune system can create hapten-specific antibodies. Phage display has been applied for hapten-specific recombinant antibody discoveries from naïve [6], immunized [7], semi-synthetic [8], and synthetic [9] libraries.

The routine practice is to immunize a mouse with one antigen and then proceed to the construction of the antibody gene library. Chickens [10] and rabbits [11,12] have, however, been successfully immunized with multiple antigens and used for recombinant antibody development. We implemented a similar strategy in this work by immunizing a mouse with different hapten-protein conjugates, thus avoiding the need for separate library constructions for each hapten.

The outcome of the selection often depends on the different selection strategies applied, and the most productive strategy for antibody discovery is difficult to predict in advance. Schier and Marks [13] demonstrated that the elution methods used in selection affect the binding properties of the antibodies. Moghaddan et al. [14] compared the affinities of anti-aflatoxin antibodies selected either with basic elutions or free aflatoxin competition and found that the free hapten elution produced antibodies with higher affinities towards aflatoxin. Various elution strategies are currently applied in the recombinant antibody selections aiming towards breakdown of the antigen-antibody interaction in the selection phase. Elution strategies can apply, for example, acid [15], base [6], free antigen [7], or the use of ultrasound [16]. It has been debated that antibodies binding with high affinity might be lost when applying competition only with free antigen or biophysical pH differences in the elution phase [17]. In order to overcome this problem, protease cleavage sites [18,19] or an additional disulphide bridge [17] between displayed antibody and phage coat protein have been used.

In order to increase the probability of obtaining antibodies for multiple haptens simultaneously, we developed robust and flexible methods utilizing four different elution strategies in parallel: acid, base, free hapten, and reducing agent elutions. The latter was used to cleave the disulphide bridge in SS-biotin linker between the hapten-protein–conjugate and magnetic beads and to release the hapten-bound antibody-displaying phages without breaking the hapten-antibody interactions. A total of five selection rounds were performed before continuing to the screening phase with high-throughput automated enzyme-linked immunosorbent assays (ELISA). Finally, the binding properties of the most promising antibody clones were analysed with Surface Plasmon Resonance (SPR).

In many cases, the same hapten-protein conjugate has been used in immunization, selection, and screening phases. This may lead to unwanted enrichment of antibodies binding to the epitopes of the carrier protein. In order to avoid this, one approach is to change the carrier proteins in the selection phase [20], and, in addition, to deplete the carrier protein binding antibodies from the phage display library by incubating the library with an excess of native carrier protein [21]. We chose to use three different carrier proteins: one in the immunization (hemocyanin), one in the selection (alkaline phosphatase (AP)), and one in the screening phase (human serum albumin (HSA)). In addition, phage display libraries were depleted in the selection phase with free AP. In this work, we demonstrate the efficiency of the selection method with two different small molecule analytes, mycophenolic acid (MPA) and ochratoxin A (OTA). Both of these haptens are mycotoxins, MPA being produced by *Penicillium* and OTA by both *Penicillium* and *Aspergillus* species. OTA is one of the most abundant mycotoxins and can be found, for example, from cereals, dried wine fruits, wine, and coffee. Human ingestion of OTA can lead to endemic nephropathy and urothelial tumours [22]. In addition, the International Agency for Research on Cancer (IARC) has classified OTA to be potentially carcinogenic to humans (Group 2B) [23]. MPA can be found, for example, in contaminated blue-veined cheese [24]. It is an immunosuppressant and effective as a drug to treat patients after organ transplantation [25].

By combining multi-immunization with a single library construction and different elution strategies in the selection phase, we were able to develop a rapid, robust, and versatile method for anti-hapten antibody discovery. Four different elution conditions in parallel utilized the potential of the library efficiently and we obtained promising antibodies against free haptens. In the case of MPA and OTA, three out of four and two out of four elution strategies, respectively, produced antibodies against free haptens, with *K*_D_ values in the range of ca. 10^−8^ M. To our surprise, in the case of MPA, the commonly used acidic elution yielded only low-affinity antibodies (*K*_D_ ≈ 10^−6^). The three high-affinity antibodies against MPA were each obtained with different elution strategies. Similar results were observed with OTA-specific antibody discovery, in which the three best antibodies were discovered by applying two different elution strategies. These results demonstrate the effectiveness of the systematic use of different elution strategies.

## 2. Results

### 2.1. Immunization and Selections

Before immunization, mycophenolic acid (MPA), ochratoxin A (OTA), and ochratoxin B (OTB) were covalently conjugated to three different carrier proteins: mollusk hemocyanin (Blue Carrier^®^, Sigma-Aldrich, St. Louis, MO, USA), human serum albumin (HSA), and alkaline phosphatase (AP). An important step in anti-hapten antibody development is verification of the success of conjugation and the conjugation degree of the hapten to carrier proteins. The use of mass spectrometer MALDI-TOF (Matrix-assisted laser desorption/ionization, time-of-flight) has been described previously [26] as being able to meet these needs, and a similar approach was used here. The MALDI-TOF results revealed that the MPA-AP and MPA-HSA had conjugation degrees of ~5 and ~29 MPA/protein, respectively. For the ochratoxins OTA-AP and OTA-HSA, conjugation degrees of ~1 and ~3 OTA/protein, and for OTB-AP and OTB-HSA conjugation degrees of ~5 and ~15 OTB/protein were obtained, respectively. The BlueCarrier™ conjugates were not analysed due to the large size of the protein [27].

Four mice were immunized with MPA- and OTB-Blue Carrier^®^ in Freund’s adjuvant. The immunoresponses were analysed from the sera of the immunized mice by ELISA using MPA- and OTB-HSA-coated microtiter wells. The ELISA responses were observed for both haptens even with 1:100,000 diluted serum samples, indicating the formation of high immune responses against both MPA and OTB. No detectable response against HSA alone was obtained [28]. The use of different carrier proteins in immunization and serum titrations efficiently excluded the false positive signals arising from the antibodies binding the carrier protein.

A recombinant antibody (fragment antigen-binding (Fab)-fragment) library derived from IgG genes of the mouse having the best immune response was essentially constructed as described in Pulli et al. [29]. Sequencing of the 40 light and 40 heavy chain variable regions of the clones from the final recombinant antibody phage display library indicated approximately 87% and 83% diversity, respectively, and the functional size of the final Fab library was ~4 × 10^8^ unique clones.

The phage display library selections were performed with magnetic bead immobilized MPA- and OTA-AP-conjugates. Although the immunization was performed with less toxic OTB-conjugates, we assumed that small differences in the chemical structures of OTA and OTB and rearrangement of heavy and light chains during the library construction would allow us to isolate OTA-specific antibodies from the final library. A total of five selection rounds were performed with varying conditions utilizing an automatic magnetic bead processor and four different elution strategies. Detailed protocols of the selection rounds are shown in Table 1. Phagemid plasmids harbouring Fab genes were isolated from enriched library pools after each selection round. Digested pools of enriched Fab genes were ligated to pKKtac expression vector [30] prior to single clone screening.

### 2.2. Antibody Screening

A total of 40 × 96 single colonies, 96 colonies from each of the five rounds, four elutions, and from two antigen selections, were screened in an automated ELISA assay performed using a Beckman Coulter robotic station. When small molecule analytes are conjugated onto carrier protein to be subsequently used in both immunization and selection, the outcome may be an unwanted enrichment of clones that recognize the carrier protein rather than the hapten epitope. In addition, enriched antibodies may show higher affinity towards hapten-protein conjugate than to hapten alone [14]. Here the antibodies binding carrier protein were successfully excluded by screening the individual recombinant antibody clones against albumin (HSA) conjugated MPA and OTA.

The antibody clones were considered to be positive when the ELISA absorbance at 405 nm was higher than 0.3 after 30 min of incubation. The distribution of obtained primary positive ELISA hits from each selection round is presented in Table 2.

A total of 78 primary positive antibodies, 70 against MPA-HSA and 8 against OTA-HSA, were found. From these, 39 primary positive antibodies against MPA-HSA and all OTA-HSA binding antibodies were chosen for further analysis. The analysis was performed with competitive ELISA in order to evaluate the binding affinities of Fab–fragments to free MPA or OTA. The competitive ELISA protocol was essentially the same as in primary screening, except diluted supernatants were mixed with varying concentrations of free MPA or OTA before transferring to the antigen coated microtiter plates. Due to the high number of clones, hapten dilution series of 1/10 was used for both haptens without replicate wells. Therefore, half-maximal inhibitory concentration (IC_50_) values are presented in Table 3 as crude ranges and not as precise inhibition concentrations. More precise IC_50_ values could be obtained by increasing the number of replicate wells and optimizing method for each antibody separately.

**Figure 1:**
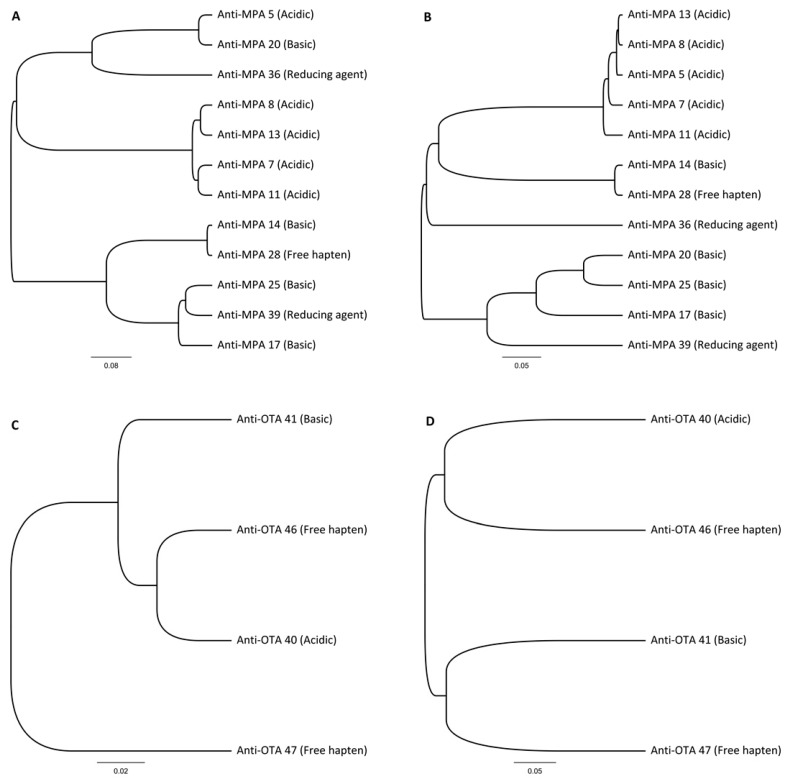
Amino acid sequences of variable regions of recombinant antibodies analysed with unweighted pair group method with arithmetic mean (UPGMA) using Geneious 6.1 software (Biomatters, Auckland, New Zealand). (**A**) Heavy chain variable regions of anti-MPA clones; (**B**) Light chain variable regions of anti-MPA clones; (**C**) Heavy chain variable regions of anti-OTA clones; (**D**) Light chain variable regions of anti-OTA clones. The elution strategy for each clone is indicated in parenthesis.

On the basis of competitive ELISA, twelve different anti-MPA and eight anti-OTA clones were further chosen for sequence analysis. The sequencing resulted in unique sequences for all twelve anti-MPA antibodies, and four unique OTA antibodies were found. Sequence homology trees of variable regions of sequenced clones are presented in Figure 1.

### 2.3. Affinity Measurements

Twelve MPA and four OTA specific antibodies as purified proteins were further analysed with SPR. The *K*_D_ values were measured with the affinity in solution by the BIAcore method [31,32,33]. The MPA or OTA-HSA conjugates were immobilized onto sensor chip surfaces. This allowed efficient regeneration of bound Fab fragments by acid injections without noticeable destruction of sensor surface binding capacity between the cycles.

**Figure 2:**
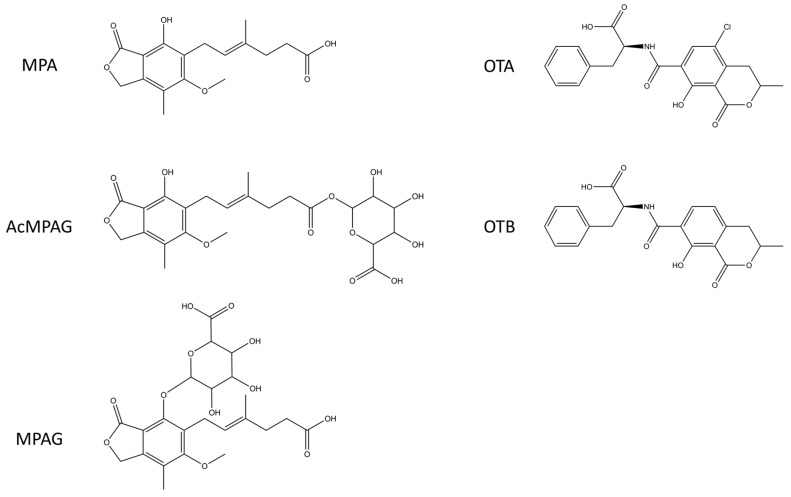
Chemical structures of the analytes used in the affinity measurements. Drawn with CambridgeSoft ChemBioDraw 12 (Perkin Elmer, Waltham, MA, USA). AcMPAG, mycophenolic acid acyl-glucuronide; MPAG, mycophenolic acid glucuronide; OTA, ochratoxin A and OTB, ochratoxin B.

In the human body, MPA is further metabolized to acyl glucuronide (AcMPAG) and phenyl mycophenolic acid glucuronide (MPAG) [34]. Since the MPA was attached from the carboxylic acid group of the hapten to the primary amines of the carrier proteins, the assumption was that the MPA specific clones would also bind to AcMPAG and that the glucuronide moiety would not create steric hindrance for the binding (Figure 2). By analogy, we also expected that the glucuronide moiety in the seventh position on the phenolic ring of MPA metabolite [35] would instead restrict the antibody binding onto the free MPAG metabolite. We tested this for the three best anti-MPA antibodies and found that all three clones bound to AcMPAG with similar affinities as to MPA, and that none of the three clones showed binding to MPAG even at 10 µM MPAG concentrations, as expected. We also found that the anti-OTA clones bound to the OTA with higher affinities than to the OTB used in the immunization phase, confirming our initial assumption that heavy and light rearrangement during library construction can be utilized in obtaining OTA-specific antibodies.

The crude affinity ranking by competitive ELISA performed from the culture supernatants had excellent correlation with the final *K*_D_ values obtained with the SPR method for purified Fab-fragments, as shown in Table 4 and Table 5.

**Figure 3:**
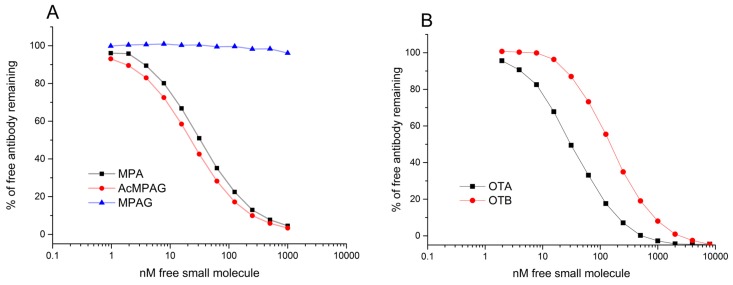
Affinity and specificity determinations of the MPA- and OTA-binding antibody clones having the highest affinities measured with SPR. (**A**) anti-MPA antibody (MPA14) competition against MPA, AcMPAG, and MPAG; (**B**) Anti-OTA antibody (OTA47) competition against OTA and OTB. Data derived from duplicates. Drawn with OriginPro 2015 (OriginLab, Northampton, MA, USA).

Interestingly, we also found that whereas MPA selections provided antibodies with affinities against free MPA ranging from 10^−8^ M to the antibodies showing no inhibition even with 10^−5^ M MPA concentrations, all the OTA-binding antibodies obtained bound to free OTA with at least 10^−7^ M *K*_D_. Affinity in solution inhibition curves of the highest affinity MPA and OTA binding antibodies are shown in Figure 3.

## 3. Discussion

By performing the immunization with multiple antigens and creating a single recombinant antibody library, the time and resources needed for separate library constructions can be significantly reduced. Antibodies were isolated for MPA used in immunization. By immunizing mice with a less toxic OTB, followed by selections and screening with OTA, we were able to obtain antibodies against the more toxic OTA [36]. The same strategy could also be applied for other toxic, harmful, or immunosuppressive small molecule analytes. To our knowledge, this is the first report describing recombinant antibody isolation for haptens from the multi-immunized mouse antibody library.

The use of different carrier proteins in immunization, selection, and screening reduces the likelihood of obtaining antibodies binding to the carrier protein alone. All the selection rounds were here studied in detail in order to gain insight into the selection process. However, in most cases, screening of only the most promising selection rounds would be sufficient. Also, other technologies, such as next-generation sequencing, could be utilized to analyse the enriched antibody libraries [37]. It has also been proposed that displaying recombinant antibody libraries in multivalent form on the first selection rounds increases the efficiency of novel recombinant antibody discovery [38].

Multiple different elution conditions were used during the selection procedure, yielding unique hapten-specific antibodies. None of the antibodies were found from the other elution strategies. Both MPA and OTA haptens had different preferences for elution conditions and only the free hapten elution method produced antibodies with *K*_D_ below 100 nM in both cases. Although acid elution is the most widely used elution method for phage rescue, no specific antibodies would have been obtained against MPA when applying only this elution strategy. High affinity IgG antibodies against MPA [39] and OTA [40] have been previously reported. To our knowledge, the antibodies described here are the highest mouse derived recombinant antibodies against these haptens. Moreover, the recombinant antibody format readily allows further affinity maturation of the obtained clones, if deemed necessary. The combination of multi-immunization, semi-automated selection with different elution modes and high-throughput screening of the individual clones with primary and competitive ELISA made the antibody development process very efficient and fast.

## 4. Materials and Methods

### 4.1. Conjugation and Matrix Assisted Laser Desorption/Ionization (MALDI)-Analysis

Mycophenolic acid (MPA) (Sigma-Aldrich, St. Louis, MO, USA), ochratoxin A (OTA) (Santa Cruz Biotechnology, Santa Cruz, CA, USA), and ochratoxin B (OTB) (Santa Cruz Biotechnology) were covalently conjugated to three different carrier proteins: mollusk hemocyanin (Blue Carrier^®^, Sigma-Aldrich), human serum albumin (HSA) (Sigma-Aldrich), and alkaline phosphatase (AP) (Sigma-Aldrich), using EDC/s-NHS chemistry according to the instructions of the manufacturer (Thermo Fisher Scientific, Waltham, MA, USA). MPA and OTB conjugates were purified from the excess of free haptens and unreacted conjugation reagents and equilibrated to PBS (20 mM sodium phosphate buffer containing 150 mM NaCl, pH 7.4) using desalting columns (NAP-5 (GE Healthcare, Piscataway, NJ, USA) or EconoPac 10DG (Bio-Rad, Hercules, CA, USA)). OTA conjugates were purified and equilibrated to PBS with Amico Ultra 10K MWCO centrifugal filters (Millipore, Bedford, MA, USA).

Matrix assisted laser desorption/ionization-time of flight mass spectrometry (MALDI-TOF MS) was applied to determine the conjugation degree of the haptens to the carrier proteins. Sinapinic acid was selected as matrix and dissolved to saturation in a 50/50 mixture of 0.1% trifluoroacetic acid (TFA) and acetonitrile. The aliquots of hapten-conjugated proteins were equilibrated to water using Amico Ultra 10K MWCO centrifugal filters (Millipore). Hapten protein conjugates in water were mixed 50/50 with the saturated matrix. One microliter (1 µL) of the mixture was spotted on the target plate and dried in air for 10 min. The analysis was made using a mass spectrometer (Brucker Autoflex II).

### 4.2. Immunization and Recombinant Antibody Library Construction

Four mice were immunized with MPA- and OTB-Blue Carrier^®^ in Freund’s adjuvant. Serum obtained at the final bleed from each mouse was analysed with ELISA against MPA-HSA, OTB-HSA, or HSA-coated wells in order to evaluate the serum responses against conjugates and carrier protein alone. An antibody phage display library derived from IgG genes of one mouse having the best immune response was essentially constructed as described in Pulli et al. [29]. Briefly, total RNA was extracted from the spleen using an RNeasy^®^ Midi–kit (Qiagen) and mRNA was copied to cDNA using a Phusion™ RT-PCR kit (Thermo Fisher Scientific). cDNA coding the IgG VHCH1 and VLCL-genes was amplified by PCR and purified from agarose gel using a Nucleospin gel extraction kit (Macherey-Nagel, Düren, Germany). Light chain pool was digested with NheI and AscI, and heavy chain pool with SfiI and NotI (all four enzymes were from New England Biolabs, Ipswich, MA, USA) and run in preparative agarose gel followed by gel extraction, as described above. Isolated digested fragments were ligated to phagemid vector [29] and transformed to *Escherichia coli* (*E. coli*) XL1-Blue electroporation competent cells (Agilent, Santa Clara, CA, USA) using electroporation. The diversity of the separate heavy and light pools was verified by sequencing variable regions of antibodies from 40 different heavy and 40 different light clones. Heavy and light chain libraries were combined together with the same restriction sites as above. The vector containing the final antibody gene library was transformed to XL1-Blue strain and the library DNA was isolated from overnight cultures of *E. coli* using a Maxi plasmid kit (Qiagen, Valencia, CA, USA).

One microgram (1 µg) of the antibody (Fab) phage display library was transformed to XL1-Blue cells in four replicate transformations using electroporation. Immediately after transformation, 3 mL of pre-warmed (+37 °C) SOC-media (0.5% yeast extract, 2% tryptone, 1 mM NaCl, 2.5 mM KCl, 10 mM MgCl_2_, 10 mM MgSO_4_, and 0.4% (*w*/*v*) glucose) was added to the transformants and incubated for 1 h at 250 rpm shaking at +37 °C. After incubation, 7 mL of pre-warmed (+37 °C) SB medium (2% yeast extract, 3% tryptone, 1% 3-(*N*-morpholino)propane sulfonic acid (MOPS), pH 7) containing 20 µg/mL carbenicillin and 10 µg/mL tetracycline was added to each separate transformation and a 50 µL sample was taken for plating. Incubations were continued with shaking at +37 °C for 1 h. From a 50 µL sample, 10-fold dilutions were made in SB medium and plated to Luria broth (LB, 0.5% yeast extract, 1% tryptone, 1% NaCl)-ampicillin plates and grown overnight at +37 °C. The carbenicillin concentration was increased to 50 µg/mL and cultivations were continued for 1 h at 37 °C with shaking. Bacteria were infected with ~10^11^ pfu of VCSM13 Helperphage (Stratagene) at +37 °C for 30 min without shaking. Volumes were raised to 100 mL with pre-warmed SB containing 50 µg/mL carbenicillin and 10 µg/mL tetracycline and growth was continued for 2 h at +37 °C with 250 rpm shaking, after which kanamycin was added to a final concentration of 70 µg/mL and the cultivations were continued at +34 °C and 300 rpm overnight.

Overnight cultures were centrifuged at 2500× *g* for 15 min and phages were precipitated from supernatants by adding 25 mL ice cold 20% PEG6000, 2.5 M NaCl to 100 mL of supernatants and incubating for 30 min at +4 °C. Precipitated phages were centrifuged at 12,500× *g* for 20 min at +4 °C and resuspended in 2 mL PBS. A second precipitation was carried out by adding 250 µL ice cold 20% PEG6000, 2.5 M NaCl to each 1 mL of resuspension, precipitating at +4 °C for 30 min, and centrifuging at 13,000× *g* for 10 min. Precipitated antibody phage display libraries were resuspended in 2 mL PBS and stored at +4 °C.

### 4.3. Antibody Selection

MPA- and OTA-AP conjugates were immobilized to magnetic beads by two different strategies: Firstly, conjugates were covalently immobilized onto epoxy coated magnetic beads (Dynabeads^®^ M-270, Thermo Fisher Scientific) according to the manufacturer’s instructions. Success of the immobilizations was verified by performing protein concentration measurements from immobilized beads (MicroBCA, Thermo Fisher Scientific). In the second strategy, the hapten-alkaline phosphatase conjugates were biotinylated with EZ-Link Sulfo-NHS-SS-Biotin (Thermo Fisher Scientific) using a 20:1 biotin-conjugate ratio following the manufacturer’s instructions, and biotinylated conjugates were purified with Amico Ultra 10K MWCO centrifugal filters (Millipore). The success of the biotinylations was verified by comparing the binding efficiency of biotinylated conjugates and native AP to streptavidin microtiter plates (Nunc). After incubation, microtiter plates were washed three times with PBS and 100 µL of 2 mg/mL *p*-Nitrophenyl Phosphate (*p*NPP, Sigma-Aldrich) in Diethanolamine-MgCl_2_ (Reagena, Toivala, Finland) was added to each well. Microtiter plates were read at 405 nm using Varioskan (Thermo Fisher Scientific) [41].

A total of five selection rounds were performed with varying conditions utilizing an automatic magnetic bead processor (KingFisher™, Thermo Fisher Scientific). Depletion of AP binding antibodies was carried out prior to each round of selection by adding 20 µg AP to the phage library pools. On the first selection round, 200 µL of precipitated phage displayed Fab library (~2.2 × 10^11^ phages) was incubated with the conjugate-coated epoxy beads or with biotinylated hapten-AP conjugates in solution. Biotinylated hapten-AP conjugates were captured from the solution with streptavidin magnetic particles (Seradyn SpeedBeads™, Thermo Fisher Scientific) prior to the washing steps. Beads were washed with 200 µL PBST (PBS + 0.05% Tween20 (Sigma-Aldrich)) and transferred to elution solution. One hundred micromolars (100 mM) of glycine-HCl pH 2.2 and 100 mM triethylamine (Sigma-Aldrich) were used for acidic and basic elutions, respectively. MPA and OTA in 1% (*v*/*v*) DMSO-PBS was used for free analyte elution. Fifty micromolars (50 mM) of DL-dithiothreitol (DTT, Sigma-Aldrich) in water was used in the elution in reducing conditions. Each elution was carried out in 100 µL volume. Detailed protocols of the selection rounds are shown in Table 1.

After the elutions, acid eluate was neutralized with 1 M Tris pH 9.6 and basic eluate with 1 M HCl. In the case of free hapten and reducing agent eluates, the phages were purified from elution mixture with Amico Ultra 10K MWCO centrifugal filters (Millipore) and equilibrated to PBS. Neutralized and buffer exchanged phage eluates were used for infection of fresh *E. coli* XL1-Blue cells (OD_600_ ≈ 1) in 1 mL SB supplemented with 10 µg/mL tetracycline. Infection was carried out at +37 °C without shaking for 15 min, after which aliquots of infected bacteria were plated to LB-ampicillin plates. After the incubations, carbenicillin and tetracycline concentrations were increased in the remaining infected bacterial cultures to 20 and 10 µg/mL, respectively, and grown in 96-well plates in a Multitron incubator shaker (INFORS) for 1 h at +37 °C at 650 rpm with 80% humidity. Thirty micrograms (30 µg) of carbenicillin and 10 µg tetracycline were added to cultures and growth was continued for 1 h. Prior to helper phage infection, 50 µL of culture aliquots were transferred to 4 mL of fresh SB containing 100 µg/mL ampicillin, 10 µg/mL tetracycline, and 1% glucose (*w*/*v*) and grown overnight at +37 °C at 225 rpm. Next, ~1 × 10^9^ pfu of helper phage VCS M13 was added to each infected 1 mL culture and incubated for 30 min at +37 °C without shaking. The helper phage infected cultures were allowed to grow at +37 °C for a further 2 h. Kanamycin was added to the cultures to a final concentration of 70 µg/mL and cultivations were carried out at +30 °C overnight. On the following day, the cultures were transferred to Eppendorf tubes and centrifuged at 10,000× *g* for 10 min. Amplified phages in the collected supernatant solutions were used without phage precipitation in the following selection rounds. Phagemid plasmids harbouring Fab genes were isolated using NucleoSpin plasmid isolation kit (Macherey-Nagel) from the 4 mL overnight cultures that were aliquoted prior to helper phage addition.

### 4.4. Primary Screening of Single Antibodies

Phagemid plasmids containing the genes of the Fab fragments from each selection round were digested with NheI and NotI restriction enzymes isolated from 1% agarose gel using a Nucleospin gel extraction kit (Macherey-Nagel) and ligated to pKKtac *E. coli* expression vector [30] containing His_6_-tag at the end of the heavy chain. Ligated products were transformed into 200 µL of *E. coli* XL1-Blue cells using the heat-shock. After transformation, the volume was increased to 600 µL with SOC and transformants were grown at +37 °C for 1 h with shaking, after which the transformants were plated onto LB-ampicillin plates.

A total of 40 × 96 single colonies, 96 colonies from each of the five rounds, four elutions, and from two antigen selections, were screened in an automated ELISA assay performed using a Beckman Coulter robotic station. Briefly, colonies were picked from agar plates to 96-wells in 100 µL of fresh SB medium containing 100 µg/mL ampicillin, 10 µg/mL tetracycline, and 1% glucose (*w*/*v*) using a QPix colony picker (Genetix, Hampshire, UK). Bacteria were grown at +37 °C at 700 rpm overnight. On the next day, cultures were inoculated into fresh SB media containing 100 µg/mL ampicillin, 10 µg/mL tetracycline, 0.1% glucose, and 1 mM isopropyl-β-d-thiogalactopyranoside (IPTG) and grown overnight at +32 °C. On the following day, 96-well induction plates were centrifuged at 3166× *g* for 15 min (Eppendorf Centrifuge 5810R). 

Three hundred nanograms (300 ng) of MPA- or OTA-HSA in 100 µL 0.1 M sodium bicarbonate buffer, pH 9.6 was coated onto Maxisorp-plates (Nunc) overnight at +4 °C. The plates were washed three times with PBST and blocked with 200 µL SuperBlock blocking buffer (Thermo Fisher Scientific). After 30 min of incubation, the plates were washed three times with PBST and 100 µL 1:10 diluted centrifuged supernatants were added to the plates. After 30 min incubation, the plates were washed as above and 100 µL of detection antibody goat anti-κ-Alkaline phosphatase (AP) (Southern Biotech; Cat. 1050-04) diluted 1:1000 in SuperBlock was added to the wells and incubated for 30 min. The plates were washed as above and 100 µL 2 mg/mL *p*NPP (Sigma) in diethanolamine-MgCl_2_ (Reagena) was added to the wells. Plates were read at 405 nm after 30 min of enzyme reaction.

### 4.5. Competitive Enzyme-Linked Immunosorbent Assay (Competitive ELISA)

Antibody clones giving absorbance 0.3 or higher at 405 nm after a 30-min enzyme reaction in the primary screening were categorized as positive hits. The competitive ELISA protocol was essentially the same as in the primary screening. Maxisorp (Nunc)-coated microtiter plates were coated with 50 ng MPA- or OTA-HSA overnight at +4 °C. After washing the plates three times with PBST, the wells were blocked with SuperBlock Blocking buffer for 30 min. Blocking solution was washed away as above. Supernatants were diluted 1:5 to the SuperBlock and mixed 50/50 with varying concentrations of free MPA or OTA in 4% DMSO-PBST. The mixtures of supernatants and free hapten were incubated for 1 h before transferring 100 µL to the microtiter plates. After 30 min of incubation, the wells were washed and detected using goat-anti-mouse-κ-AP as in the primary screening.

### 4.6. Protein Purification

A total of 16 clones, 12 against MPA and 4 against OTA, were selected on the basis of competitive ELISA and purified using standard immobilized metal affinity chromatography (IMAC) with Cu^2+^. Briefly, batches of pKKtac production vector containing the selected Fab-His_6_ clones were transformed to *E. coli* RV308 production strain (ATCC 31608) and plated onto LB-amp plates. Single colonies were picked into 4 mL of SB medium containing 100 µg/mL ampicillin and 1% glucose and grown at +37 °C and 225 rpm overnight. On the next day, the cultures were inoculated into 100 mL TB medium (1.2% yeast extract, 2.4% soy peptone, 55 mM K_2_HPO_4_, 17 mM KH_2_PO_4_, 5.2% glycerol) containing 100 µg/mL ampicillin and grown at +37 °C to OD_600_ ~ 2.5. Antibody production was induced by adding IPTG to a final concentration of 1 mM. The temperature was decreased to +30 °C for the production phase and cells were harvested and supernatants collected on the following day. Antibodies were purified from the culture supernatant by metal affinity chromatography according to the manufacturer’s instructions using an increasing concentration of imidazole (Sigma-Aldrich) for elution [42]. Dialysis was used to equilibrate the antibodies to PBS buffer before characterization of the binding properties. Purity of each of the proteins was evaluated with SDS-PAGE protein gel [43] and concentrations were calculated using theoretical extinction coefficients at 280 nm (ExPASy ProtParam tool, http://web.expasy.org/protparam/) obtained from the sequencing data.

### 4.7. Affinity Measurements

Affinities of the purified Fab fragments were analysed using a surface plasmon resonance instrument BIAcore T200 (GE Healthcare) by the affinity in solution method [31,32,33]. MPA- or OTA-HSA was immobilized onto CM5 chips using EDC/s-NHS chemistry following the manufacturer’s instructions. One channel was left blank to serve as a reference. Purified Fab fragments were diluted into PBS-P+ (PBS, 0.05% P20) (GE Healthcare) supplemented with 2% DMSO (*v*/*v*) (Sigma-Aldrich) followed by three-quarters dilution series in the same buffer in order to create a calibration series for free Fab. A total of 10 dilutions were used in each assay. Fab fragments were pre-incubated with varying concentrations of free MPA, AcMPAG, MPAG, OTA, or OTB in 2% DMSO-PBSP. Concentrations of remaining free Fab-fragments in the solutions were analysed against immobilized MPA- or OTA-HSA surface using a contact time of 300 s, dissociation for 60 s, and a flow rate of 10 µL/min. Regeneration of bound Fab-fragments from the MPA- and OTA-HSA surface was achieved with 60 s and 180 s pulses of 10 mM Glycine-HCl, pH 1.5 (GE Healthcare), respectively. DMSO standard curves were applied in order to tolerate minor DMSO concentration variations between samples. Data collection point was set to be 10 s after the end of the injection [33]. Data was analysed using BIAcore T200 Evaluation Software with four-parameter logistic function. All MPA binding clones were first analysed with a constant Fab concentration of 30 nM. For the three anti-MPA clones having the highest affinities, the concentrations of Fabs were further decreased to 2 nM. OTA-binding antibodies were all analysed with constant 3 nM Fab concentrations.

## Figures and Tables

**Table 1:** A detailed summary of variables in the conditions of the selection rounds. Fab, fragment antigen-binding.

Capture					Elution			
Selection Round	Fab Antibody Library	Amount of Magnetic Beads ^a^	Amount of Biotinylated Conjugate ^b^	Time	Washing Time	Release Time ^a^	Release Time ^b^	Free Hapten Concentration in Elutions
**1st**	200 µL precipitated library	5 µL	1.5 µg	overnight	6 × 20 s	20 min	1 h	100 nM
**2nd**	200 µL non-precipitated library	4 µL	1.5 µg	1 h	5 × 20 s	20 min	1 h	10 nM
**3rd**	200 µL non-precipitated library	3 µL	1.0 µg	30 min	5 × 40 s	20 min	1 h	1 nM
**4th**	200 µL non-precipitated library	2 µL	0.5 µg	15 min	5 × 2 min	20 min	1 h	100 pM
**5th**	200 µL non-precipitated library	1 µL	0.1 µg	15 min	5 × 10 min	20 min	1 h	10 pM

^a^: acid, base and free hapten elutions; ^b^: reducing agent elutions.

**Table 2:** Number of obtained positive ELISA hits from primary screening. MPA, mycophenolic acid; OTA, ochratoxin A.

Selection		Primary Positive Clones					Chosen for Competitive ELISA/Total Hits
		Round					
Antigen	Elution Condition	1	2	3	4	5	
**MPA**	Acidic	2	2	4	4	8	13/20
	Basic	1	4	8	8	9	12/30
	Free Hapten	1	0	0	0	4	4/5
	Reducing agent	3	6	1	0	5	10/15
**OTA**	Acidic	0	1	0	0	0	1/1
	Basic	0	0	0	2	3	5/5
	Free Hapten	0	0	0	0	2	2/2
	Reducing agent	0	0	0	0	0	0/0

**Table 3:** Distribution of the competitive ELISA results and corresponding elution conditions.

Antigen	Elution Condition	IC_50_			
		<100 nM	0.1–1 µM	1–10 µM	>10 µM
**MPA**	Acidic	0	0	4	9
	Basic	1	0	0	11
	Free hapten	1	0	0	3
	Reducing agent	1	0	2	7
**OTA**	Acidic	1	0	0	0
	Basic	0	1 ^a^	0	0
	Free hapten	2	0	0	0
	Reducing agent	0	0	0	0

^a^: Of the eight sequenced anti-OTA clones, five clones obtained from basic elutions had identical amino acid sequences.

**Table 4:** Affinities and specificities of the anti-MPA antibodies measured by competitive ELISA and Surface Plasmon Resonance (SPR).

Anti-MPA Clone	Competitive ELISA against MPA, IC_50_ (nM)	*K*_D_ from SPR Measurements (nM)		
		MPA	AcMPAG	MPAG
**14**	<100	39	45	>10,000
**28**	<100	40	55	>10,000
**36**	<100	87	52	>10,000
**11**	1000–10,000	1300		
**5**	1000–10,000	1400		
**8**	1000–10,000	1400		
**7**	1000–10,000	1600		
**13**	1000–10,000	2800		
**20**	1000–10,000	8300		
**17**	>10,000	>10,000		
**25**	>10,000	>10,000		
**39**	>10,000	>10,000		

**Table 5:** Affinities and specificities of the anti-OTA antibodies measured by competitive ELISA and SPR.

Anti-OTA Clone	Competitive ELISA against OTA, IC_50_ (nM)	*K*_D_ from SPR Measurements (nM)	
		OTA	OTB
**47**	<100	34	162
**46**	<100	42	114
**40**	<100	157	346
**41**	100–1000	447	3254

## Abbreviations

Fab	Fragment antigen-binding
HSA	Human serum albumin
AP	Alkaline phosphatase
